# A Structural Basis for IκB Kinase 2 Activation Via Oligomerization-Dependent *Trans* Auto-Phosphorylation

**DOI:** 10.1371/journal.pbio.1001581

**Published:** 2013-06-11

**Authors:** Smarajit Polley, De-Bin Huang, Arthur V. Hauenstein, Amanda J. Fusco, Xiangyang Zhong, Don Vu, Bärbel Schröfelbauer, Youngchang Kim, Alexander Hoffmann, Inder M. Verma, Gourisankar Ghosh, Tom Huxford

**Affiliations:** 1 Department of Chemistry & Biochemistry, University of California–San Diego, La Jolla, California, United States of America; 2 Structural Biochemistry Laboratory, Department of Chemistry & Biochemistry, San Diego State University, San Diego, California, United States of America; 3 Signaling Systems Laboratory, University of California–San Diego, La Jolla, California, United States of America; 4 Argonne National Laboratory, Argonne, Illinois, United States of America; 5 Laboratory of Genetics, Salk Institute for Biological Studies, La Jolla, California, United States of America; Brandeis University, United States of America

## Abstract

Activation of the IκB kinase (IKK) is central to NF-κB signaling. However, the precise activation mechanism by which catalytic IKK subunits gain the ability to induce NF-κB transcriptional activity is not well understood. Here we report a 4 Å x-ray crystal structure of human IKK2 (hIKK2) in its catalytically active conformation. The hIKK2 domain architecture closely resembles that of *Xenopus* IKK2 (xIKK2). However, whereas inactivated xIKK2 displays a closed dimeric structure, hIKK2 dimers adopt open conformations that permit higher order oligomerization within the crystal. Reversible oligomerization of hIKK2 dimers is observed in solution. Mutagenesis confirms that two of the surfaces that mediate oligomerization within the crystal are also critical for the process of hIKK2 activation in cells. We propose that IKK2 dimers transiently associate with one another through these interaction surfaces to promote *trans* auto-phosphorylation as part of their mechanism of activation. This structure-based model supports recently published structural data that implicate strand exchange as part of a mechanism for IKK2 activation via *trans* auto-phosphorylation. Moreover, oligomerization through the interfaces identified in this study and subsequent *trans* auto-phosphorylation account for the rapid amplification of IKK2 phosphorylation observed even in the absence of any upstream kinase.

## Abstract 2

Conformational change in human IKK2 permits dimers to form higher-order oligomers that support interaction between kinase domains and promote activation through *trans* auto-phosphorylation.

## Author Summary

IκB kinase (IKK) is an enzyme that quickly becomes active in response to diverse stresses on a cell. Once activated, IKK promotes an array of cellular defense processes by phosphorylating IκB, thereby promoting its degradation and liberating its partner, the pro-survival transcription factor NF-κB; NF-κB is then free to relocate to the nucleus where it can modulate gene expression. Our X-ray crystallographic studies on an active version of the human IKK2 isoform reveal that the enzyme adopts a unique open conformation that permits pairs of IKK2 enzymes to form higher order assemblies in which their catalytic domains are in close proximity. Disruption of IKK2's ability to form these assemblies, by introducing changes that interfere with the surfaces that mediate oligomerization, results in IKK2 enzymes that are greatly impaired in their ability to become activated in cells. We propose that after oligomerization the neighboring catalytic domains then phosphorylate each other as part of the activation process. Our findings also suggest that targeted small molecules might disrupt cell survival by blocking IKK2 assembly in cells.

## Introduction

The IκB kinase (IKK) earns this name for its ability to phosphorylate two serine residues positioned near the N-terminal end of cytoplasmic IκB inhibitor proteins. IKK activity, which is induced by multiple cell stress and inflammatory signals and which quickly leads to ubiquitin-dependent proteolysis of IκB, is central to the rapid induction of transcription factor NF-κB and the elevated expression of NF-κB target genes [1],[2]. The prototypical IKK activity purifies from TNF-α–treated cells as a complex consisting of three necessary subunits: IKK1/IKKα, IKK2/IKKβ, and NEMO/IKKγ (referred to in this report as IKK1, IKK2, and NEMO, respectively) [3]–[6]. While IKK1 and IKK2 are closely related kinase domain (KD)-containing enzymes, NEMO is an obligate scaffolding protein that exhibits higher affinity for IKK2 than IKK1 [6]–[10]. A catalytically active 700–900 kDa complex containing all three subunits is the most abundant form of IKK in stimulated cells. However, both IKK1 and IKK2 homodimers have also been observed [11]. Gene knockout studies in mice have revealed that the IKK2 subunit is primarily responsible for inducing NF-κB activity through signal-dependent phosphorylation of cytoplasmic IκB proteins (IκBα, IκBβ, and IκBε) and p105/NF-κB1 [8],[12],[13].

Despite the fact that IKK occupies the central position in brokering cellular responses to diverse stress signals through NF-κB, the precise biochemical mechanism behind IKK activation remains incompletely understood. One essential feature of IKK activation is its phosphorylation at two activation loop serine residues (S177 and S181 in hIKK2). Mutation of both these residues to alanine impedes activation while glutamic acid substitutions render the kinase constitutively active [4],[14]. It has been shown that K63-linked and/or linear ubiquitin chain formation is required for IKK activation by some inducers such as IL-1 and Toll-like receptor agonists [15]. NEMO appears to link IKK2 to upstream signaling complexes by interacting with a C-terminal portion of IKK2 and with poly-ubiquitin chains through its N-terminal and central coiled-coil regions, respectively [10],[16]–[18]. Moreover, several additional kinases, such as TAK1, have been implicated in IKK activation [19]. Apart from phosphorylation by upstream kinases, however, IKK2 auto-phosphorylation has also been suggested as a means of activating IKK2 [20]. For example, Xia et al. recently showed that unanchored poly-ubiquitin chains can promote IKK activation independent of any upstream kinase [21].

The recently published 3.6 Å X-ray crystal structure of a highly conserved IKKβ from *Xenopus laevis* (xIKK2) revealed for the first time the domain organization of a catalytic IKK subunit, albeit in an inactivated form that shed little light on its mechanism of activation [22]. Here we report the 4.0 Å X-ray crystal structure of Human IKK2 (hIKK2) in a unique open conformation that presents its KD in a catalytically active conformation. Pairs of hIKK2 dimers pack against one another throughout the crystal to generate several crystallographically distinct versions of the same higher order oligomer. Introduction of mutations within the surfaces that mediate this oligomerization disrupts hIKK2 activation loop phosphorylation in cells, suggesting that IKK2 activation requires assembly of higher order homo-oligomers for *trans* auto-phosphorylation.

## Results

### The Human IKK2 X-Ray Crystal Structure

**Figure 1:**
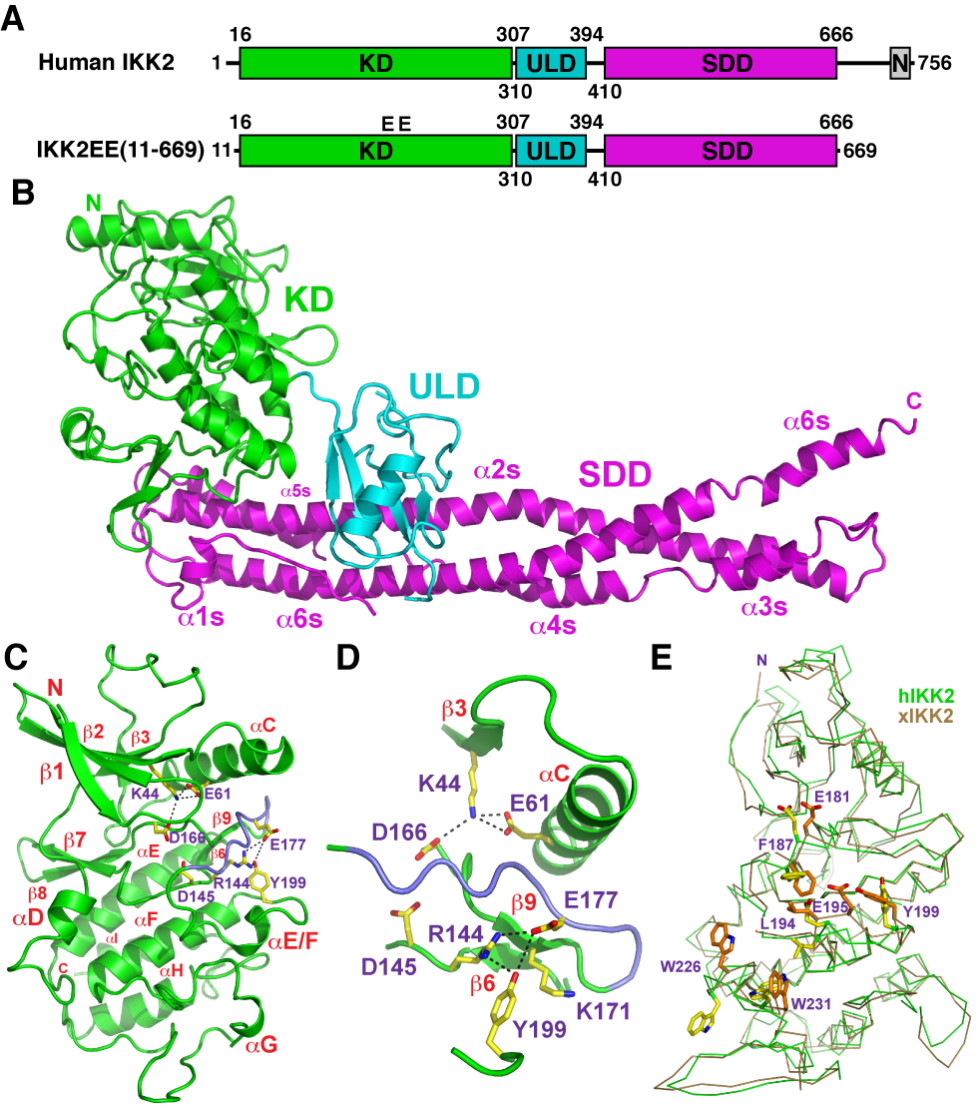
The hIKK2 X-ray crystal structure. (A) Domain organization schematics of full-length hIKK2 (above) and crystallized protein construct (below). The KD, ubiquitin-like domain (ULD), scaffold dimerization domain (SDD), and NEMO-binding region (N) are indicated and the domain borders are numbered. “EE” represents mutation of two active site serines 177 and 181 to glutamic acid residues in the crystallized protein. (B) Ribbon diagram representation of the hIKK2 X-ray crystal structure. Coloring and labels correspond to part A. Individual helices of the SDD are labeled as are the N and C termini (N and C, respectively). (C) Ribbon diagram representation of the hIKK2 KD with secondary structure elements and key amino acid side chain positions labeled. (D) Close-up view of the structural elements and amino acid residues immediately surrounding the activation loop (blue). (E) Superposition of hIKK2 (green) and xIKK2 (brown) KDs depicted in Cα-trace representation. Several amino acid residues that adopt significantly different positions in the two structures are rendered as sticks (yellow for hIKK2; orange for xIKK2) and labeled.

We determined the X-ray crystal structure of a nearly full-length (amino acids 11–669), constitutively active (S177E/S181E) hIKK2 from a tetragonal space group by molecular replacement and Deformable Elastic Network (DEN)-assisted refinement (Figure 1A) [23]. The final model has a crystallographic *R*-factor of 26.7% and free *R*-factor of 29.9% (Table 1). hIKK2 adopts an elongated shape composed of three distinct folded domains: an N-terminal KD, a central ubiquitin-like domain (ULD), and a C-terminal scaffold dimerization domain (SDD) (Figure 1B). The structures of the individual domains and their relative arrangement closely resemble that observed in the xIKK2 crystal structure [22]. This is not surprising as the two proteins share 74% sequence identity throughout the region crystallized (Figure S1).

**Table 1:** Data collection and refinement statistics

Data Collection and Refinement	IKK2(11–669)EE
*Data collection*	
X-ray source	APS 19ID
Wavelength (Å)	0.97935
Space group	P4_1_22
Unit cell (Å)	
a	170.81
b	170.81
c	509.56
Molecules/asymm. unit	6
Resolution range (Å)^a^	30.0–3.98 (4.12–3.98)
*R*_sym_ (%)	13.9 (73.6)
Observations	248,094
Unique reflections	63,535
Completeness (%)	96.2 (97.9)
<*I*/σ>	6.3 (2.4)
*Refinement*	
Number of reflections^b^	53,076
*R*_cryst_ (%)	26.7 (37.6)
*R*_free_ (%)^c^	29.9 (39.3)
Protein atoms	30,060
R.m.s.d.	
Bond lengths (Å)	0.0093
Bond angles (°)	1.08
Ramachandran plot	
Favored	65%
Allowed	33%
Disallowed	2%
MolProbity score^d^	3.40
PDB accession code	4E3C

a Data in parentheses are for highest resolution shell.

b Reflections with |*F*_O_|/σ<1.0 rejected.

c Calculated against a cross-validation set of 3.8% of data selected at random prior to refinement.

d Combines clashscore, rotamer, and Ramachandran evaluations in to a single score, normalized to the same scale as x-ray resolution [40].

The KD exhibits all of the sequence and structural features typical of a functional catalytic KD in its active conformation [24]. Residues from the catalytic loop, the phosphate binding site, and the magnesium binding loop occupy the positions of an ordered catalytic center that can catalyze the phosphoryl transfer reaction (Figure 1C). For example, the K44–E61 pair contributed by beta-strand β3 and alpha-helix αC is properly oriented to form a salt bridge, the DFG tripeptide residues of the Mg^2+^ binding loop (DLG in IKK2) occupy their “active” positions, the catalytic base D145 is poised for catalysis, and the beta-sheet formed by strands β6 and β9 contains three hydrogen bonds, a signature feature of all active kinases (Figure 1D). Furthermore, the KD within the hIKK2 crystal structure also exhibits the constellation of buried hydrophobic residues that form the “spine” of an active protein kinase (Figure S2) [25].

Comparison of the KD from hIKK2 and xIKK2 reveals notable differences in their active site conformations. The β6–β9 sheet is shifted in xIKK2 and the conformation of leucine within the DLG motif is considerably altered. Consequently, the regulatory hydrophobic spine of xIKK2 is broken. Furthermore, side chain conformations of several other residues exhibit clear differences (Figure 1E). It is possible, therefore, that inhibitor binding by xIKK2 induces conformational changes within the activation segment that lead to the observed inactivated conformation of the otherwise constitutively active xIKK2 KD.

An extended polypeptide stretch spans amino acid residues 391 and 410, a dimension of roughly 40 Å, and links the ULD to the SDD, a C-terminal alpha-helical domain that mediates dimerization. The SDD consists primarily of two long alpha-helices (α2s and α6s), each approximately 115 Å in length, that run parallel to one another and a third alpha-helical segment extending roughly the same distance to complete the anti-parallel three-helix bundle. However, this third stretch is discontinuous, being composed of alpha-helices α3s, α4s, and α5s. It is also the most solvent exposed of the three long alpha-helical passes.

**Figure 2:**
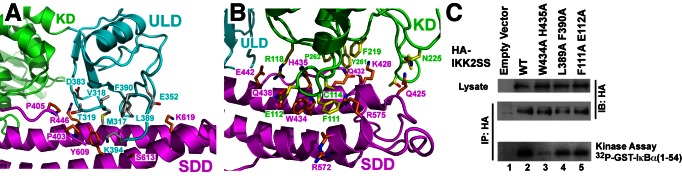
Domain–domain interactions in IKK2. (A) Close-up view of the ULD–SDD interface. Coloring is consistent with Figure 1. (B) Close-up view of the KD–SDD interface. (C) In vitro kinase assays in which hIKK2 with mutations that target interdomain interfaces (lanes 3–5) is compared against the native protein (lane 2).

One of the signature structural features of IKK2 is the dependence of KD function upon its two more C-terminal domains [22],[26]. The hIKK2 KD contacts the ULD burying 360 Å^2^ total surface area. The similarities at their interface and in their relative orientations in both the xIKK2 and hIKK2 structures suggest that the IKK2 KD and ULD function together as a single rigid structural and functional unit. The IKK2 structure reveals extensive intramolecular interactions between the KD–ULD and SDD. Each module buries nearly 1,200 Å^2^ of surface area, involving 11 residues from the KD–ULD and another 11 from the SDD (Figure 2A,B). In order to assess the importance of IKK2 interdomain interactions on catalytic activity, we prepared three double mutant HA-IKK2 transfection plasmids. Unless indicated otherwise, all hIKK2 mutations employed in this study were introduced against the background of the native (S177/S181) sequence. One pair of mutations, F111A/E112A, targets the KD at its interface with the SDD, while two others, L389A/F390A and W434A/H435A, map to the ULD and SDD, respectively. The IKK2 W434A/H435A mutant exhibited the greatest defects when immunoprecipitated and assayed for in vitro IκBα phosphorylation activity, while IKK2 L389A/F390A and F111A/E112A mutants proved only partially defective (Figure 2C). Although the detailed mechanism by which interdomain interactions serve to tune kinase activity and specificity remains to be determined, this experiment illustrates that disrupting articulation between the three domains is detrimental for IKK2 function.

### The hIKK2 Dimer Interface

**Figure 3:**
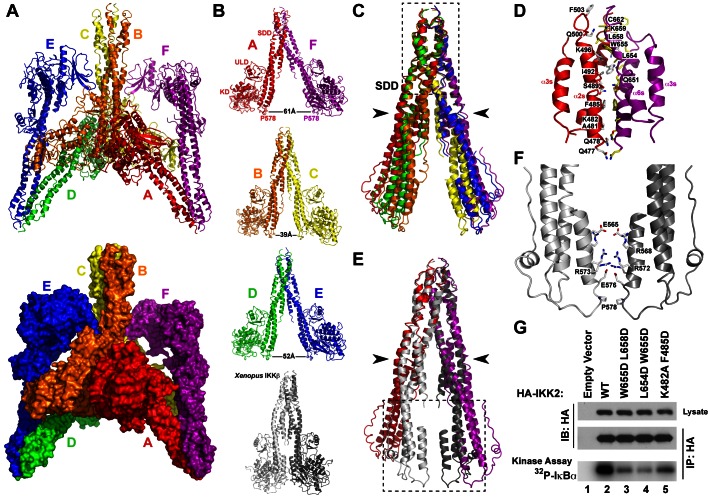
The hIKK2 dimer interface. (A) Ribbon diagram (top) and space filling (bottom) representations of six hIKK2 protomers in the asymmetric unit. The individual subunit chains are labeled A–F and rainbow colored. (B) Ribbon diagrams of three hIKK2 dimers taken from the asymmetric unit. The distances between P578 residues from each hIKK2 in the dimers are labeled. For comparison, the xIKK2 crystal structure observed is shown below in its relatively closed conformation. (C) Overlay of the SDD from six monomers in the hIKK2 asymmetric unit. The three dimers overlay perfectly at their SDD distal ends (inside dashed box). Movement about a hinge point (black arrowheads) results in the differences observed in the portions of the SDD more proximal to the KD. (D) A close-up view reveals the series of hydrophobic and ionic interactions that mediate the dimer interface. (E) Superposition of the A:F dimer SDD from the hIKK2 structure and the xIKK2 dimer. (F) Close-up view of the boxed region from panel D reveals additional interactions present within the interface of the xIKK2 structure SDD. (G) In vitro kinase assays monitoring the activity of immunoprecipitated HA-IKK2 toward IκBα. Mutations targeting the dimer interface (lanes 3–5) are compared against the wild-type protein (lane 2).

The hIKK2 X-ray crystal structure contains six distinct protomers within the crystallographic asymmetric unit. Each mediates contact with three neighbors to form a network of interactions between subunits (Figure 3A). As IKK2 is known to function as a component of a high molecular weight complexes in cells and since surfaces of enzymes that participate in crystal contacts have often later been discovered to mediate physiologically relevant binding reactions, it is critical to identify whether the observed interactions between protomers in the hIKK2 crystal structure are involved in the process of IKK activation in vivo.

The principal interaction that mediates contacts between hIKK2 protomers in the X-ray crystal structure is created exclusively by one face of the C-terminal portion of the SDD (Figure 3B). A series of hydrophobic residues from alpha-helices α2s and α6s on each protomer pack symmetrically against one another to form the dimer. This hydrophobic core is further stabilized by ionic bonds creating an interface that buries roughly 1,250 Å^2^ per subunit. Each of six hIKK2 subunits (chains A through F) in the asymmetric unit participates in this dimer interface: chain A with F; B with C; D with E. An analogous surface contributes to the dimer interface observed in the xIKK2 structure. In contrast to the *Xenopus* model, however, the N-terminal KD–ULD portions in each of the three hIKK2 dimers splay apart at a distance of between 39 and 61 Å from one another.

Overlay of the three hIKK2 dimers reveals near perfect superposition at the distal portion of the SDD where the two subunits associate (Figure 3C,D). Similarly, superposition of hIKK2 (chains A and F) upon the xIKK2 dimer confirms that their dimer interfaces are nearly identical at the distal end of the SDD but differ significantly at the end more proximal to the KD–ULD (Figure 3E). The difference can be attributed to a hinge-like movement within the SDD near residue G525 between alpha-helices α4s and α5s. This pivot point allows the more N-terminal portions of hIKK2 to swing away from one another. Residues that are observed to mediate dimerization more proximal to the KD–ULD portion in the xIKK2 structure are primarily polar/charged, suggesting that this segment might be able to alter its conformation from the nearly parallel closed conformation observed in the xIKK2 crystal structure to the open conformation of hIKK2 (Figure 3F).

As the dimer interface within the distal portion of the SDD is common to all the dimers in both the hIKK2 and xIKK2 structures, it can be concluded with some certainty that both enzymes dimerize through this region. Xu et al. have validated the functional importance of this interface in constitutively active (S177E/S181E) human IKK2 by preparing enzymes with pairs of mutations that disrupt dimerization and finding that they are primarily monomeric in solution [22]. We further tested the functional consequences of destabilizing this region by altering three pairs of residues (W655D/L658D, L654D/W655D, and K482A/F485D) in human IKK2 (native activation loop sequence) and measuring the kinase activity of these mutant enzymes in vitro. After HA–IKK2 native and mutant proteins were immunoprecipitated from transfected HEK293T cells, a significant decrease in IKK2 catalytic activity was observed in the mutants (Figure 3G). These results suggest that amino acid residues from the SDD that mediate the dimer interface are important in hIKK2 activation and/or IκBα phosphorylation.

### Higher Order hIKK2 Assembly

**Figure 4:**
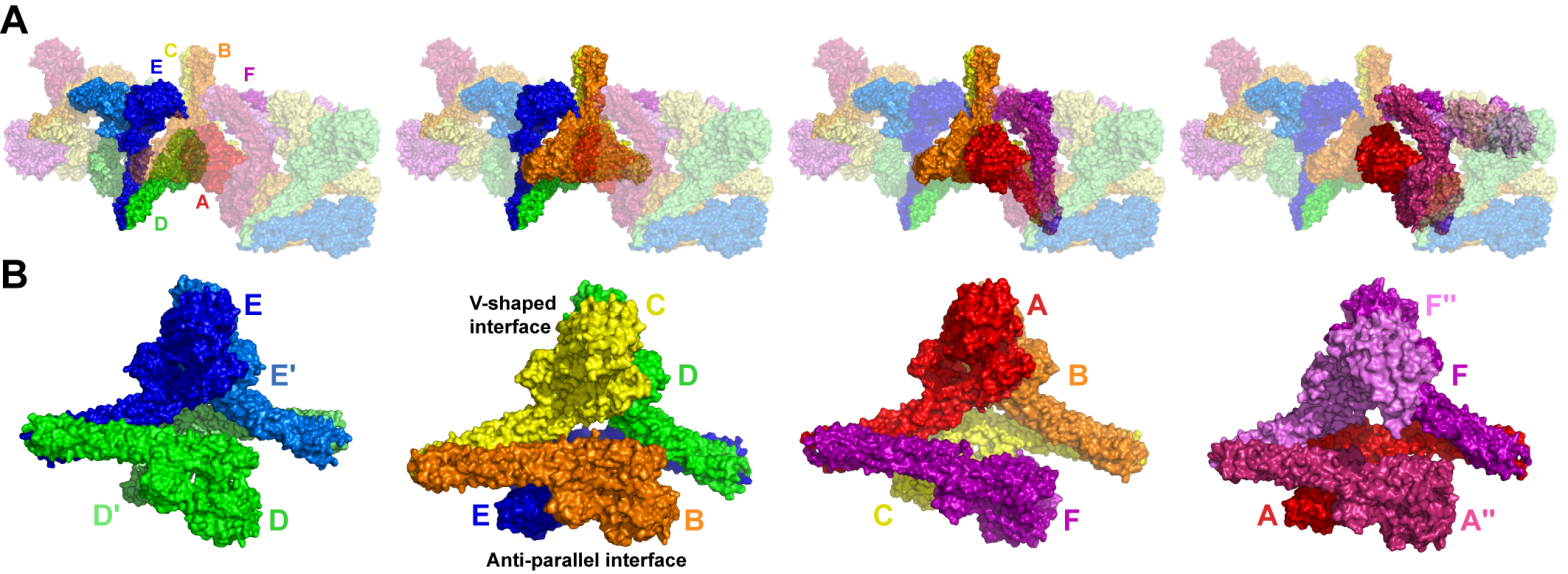
Oligomerization of hIKK2 dimers in the crystal. (A) Three successive asymmetric units, each composed of six protomers, are taken from the hIKK2 X-ray crystal structure and depicted with the center asymmetric unit (labeled A–F) and colored with surface rendering as in Figure 3A. Within this arrangement can be found four unique but closely related versions of the same dimer of dimers. Beginning from the left, there are four protomers (D, E, and another D′ and E′ from the symmetry-related asymmetric unit) that assemble into a tetramer. These four polypeptide chains are depicted as opaque, while the remaining protomers are rendered as semitransparent. In the second depiction of the same assembly of three asymmetric units, chains B, C, D, and E are rendered opaque and reveal themselves to assemble with a similar tetrameric arrangement. Likewise, protomers A, B, C, and F in the third panel and A, F, and the symmetry-related A″ and F″ chains assemble into renditions of the same tetramer. (B) Close-up views of the four unique assemblies viewed perpendicular to their 2-fold rotation axes reveal their close similarity.

A close inspection of the arrangement of hIKK2 within the crystal reveals that individual dimers stack upon neighbors to form a continuous repeating oligomeric chain (Figure 4). Oligomerization results from the asymmetric interaction of neighboring dimers such that the N-terminal KD–ULD portions and proximal SDD of two protomers interact to form a bent “V-shaped” interface, while the other two protomers rest next to one another in a nearly antiparallel arrangement involving the KD, ULD, and central portion of the SDD. Slight variations in these interfaces, due likely to the influence of crystal packing forces, introduce small breaks in the otherwise continuous chain of similarly stacked dimers. This results in a crystal composed of four distinct but structurally similar “tetramers” (chains F-A-B-C, B-C-D-E, D-E-E′-D′, and A″-F″-F-A, where the prime and double-prime symbols indicate chains from two different neighboring asymmetric units), while the fundamental asymmetric unit of the crystal contains three hIKK2 homodimers (Figure 4B). A significant portion of the hIKK2 surface is involved in mediating its oligomerization with its neighbors in the crystal. The V-shaped interfaces (calculated from chains F-A-B-C, B-C-D-E, and D-E-E′-D′) bury roughly 2,000 Å^2^ of solvent exposed surface area on average, while each antiparallel interface (calculated from chains F-A-B-C, B-C-D-E, and A″-F″-F-A) excludes nearly 1,200 Å^2^.

Prior to solving and refining the hIKK2 crystal structure from the tetragonal space group, we obtained complete X-ray diffraction data to 7 Å resolution on a constitutively active (S177E/S181E) hIKK2 construct encompassing amino acids 1–700 crystallized in a hexagonal space group (Figure S3A). Molecular replacement using the refined hIKK2EE(11–669) model as a probe yielded a probable solution to the hexagonal crystal structure in which open hIKK2 dimers employ their V-shaped and antiparallel oligomerization interfaces to stack against crystallographically identical dimers in a continuous linear array throughout the crystal unit cell (Figure S3B). The crystal packing for this solution is ideal and maximum likelihood rigid body refinement of the resulting model against all data between 50 and 7 Å results in a significant decrease in *R*-factors (Table S1).

**Figure 5:**
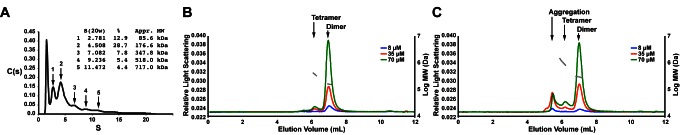
Evidence for oligomerization of hIKK2 dimers in solution. (A) Analytical ultracentrifugation sedimentation velocity experiments on concentrated samples of full-length hIKK2 reveal a pattern that correlates with monomer–dimer equilibrium as well as formation of tetramers, hexamers, and octamers in solution. Arrows mark peaks and a summary of data output including calculated molecular weights is inset. (B) When full-length, ATP-treated hIKK2 is analyzed by SEC-MALLS, one observes peaks that correspond to dimer (major peak) and tetramer (minor peak). (C) The hIKK2 I413A/L414A mutant protein displays defects in its ability to undergo reversible dimer–tetramer transitions without aggregating.

In light of the common interactions observed to mediate contacts between molecules in the two hIKK2 crystal forms, we hypothesized that oligomerization through these surfaces could play a role in IKK function. In order to test this structure-based hypothesis, we first investigated whether hIKK2 assembles spontaneously into higher order oligomers in solution. Analytical ultracentrifugation sedimentation velocity experiments carried out on His-tagged full-length hIKK2 identified monomer and dimer populations. Moreover, peaks corresponding in size to tetramers, hexamers, and even octamers could also be clearly detected (Figure 5A). In a separate experiment, size exclusion chromatography followed by simultaneous measurement of absorbance, multi-angle laser light scattering, and refractive index (SEC-MALLS) to allow for shape-independent molecular weight determination of hIKK2 pretreated with ATP and concentrated as for crystallization clearly detected dimers and tetramers (Figure 5B). The proportion of tetramer to dimer remained relatively small even as the concentration approached the solubility limit of the protein, suggesting that spontaneous and reversible assembly of hIKK2 dimers into higher order oligomers is a low-affinity process in vitro. SEC-MALLS further revealed that the double mutation of residues I413 and L414, both of which reside at the V-shaped interface, to alanine disrupts the smooth transition of hIKK2 from dimer to tetramer such that significantly more of the mutant protein elutes as a heterogeneous aggregate (Figure 5C).

**Figure 6:**
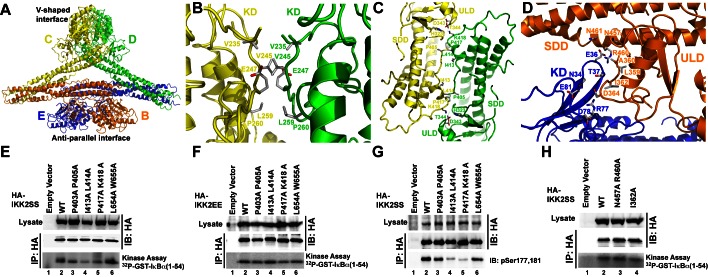
Oligomerization of hIKK2 dimers. (A) Ribbon diagram of the interaction between two neighboring hIKK2 dimers in the crystal. Their asymmetric association gives rise to two unique intersubunit interfaces. (B) Close-up view of residues that interact between the KDs at the V-shaped interface. (C) Additional residues that mediate V-shaped interface interactions between the ULD an SDD. (D) Close-up view of interacting residues within the anti-parallel interface. (E) In vitro kinase assay reveals that catalytic activity of hIKK2 with mutations that disrupt the V-shaped interface (lanes 3–5) is drastically reduced compared to wild-type protein (WT-lane 2). (F) In vitro kinase assays with the same WT mutant proteins in which activation loop serines are mutated to glutamate. (G) Immunoblotting with anti-phospho-Ser177,181 antibody reveals that the decrease in catalytic activity observed in the V-shaped interface mutants correlates with activation loop phosphorylation status. (H) In vitro kinase assays reveal the modest effects on hIKK2 catalytic activity of mutation at the antiparallel interface.

The relatively low-affinity oligomerization observed in vitro does not preclude the possibility that transient interactions between hIKK2 dimers might be involved in the transition of the enzyme from its inactive to active state in vivo. In order to test whether the oligomerization interfaces observed in the hIKK2 X-ray crystal structure are critical to its biochemical function, we next engineered additional mutations aimed at destabilizing either the V-shaped or antiparallel interfaces and assayed their in vitro kinase activity after immunoprecipitation from transfected HEK293T cells (Figure 6). Three hIKK2 double mutants designed to disrupt the V-shaped interface, P403A/P405A, I413A/L414A, and P417A/K418A, displayed a significant decrease in their ability to function as catalysts of IκBα phosphorylation in vitro relative to native protein or proteins in which residues at the dimer interface were mutated (Figure 6E). This decreased catalytic efficiency could result either from disruption of the kinase phospho-transfer mechanism or by interfering with the ability of the kinase to become activated. In order to distinguish whether the V-shaped mutations affect kinase *activity* or kinase *activation*, we next carried out the same assay with hIKK2 constructs in which the mutations were introduced against the constitutively active (S177E/S181E) hIKK2 background. These constitutively active proteins bearing mutations at their V-shaped oligomerization interface functioned with kinase activity identical to the native sequence (Figure 6F). Moreover, immunoblot of the hIKK2 proteins bearing mutations only at the V-shaped interface with a monoclonal antibody raised to specifically recognize phosphorylated S177 and S181 on hIKK2 revealed that these activation loop residues were not phosphorylated upon overexpression (Figure 6G). These experiments strongly suggest that residues observed to mediate oligomerization through the V-shaped interface in the hIKK2 crystal structure are critical in the transition of the kinase from its inactive to active state. Similar experiments aimed to assess the role of the antiparallel interface in IKK2 function indicated that mutations that target this surface do not affect kinase activation (Figure 6H).

### KD–KD Interactions of Oligomeric hIKK2

**Figure 7:**
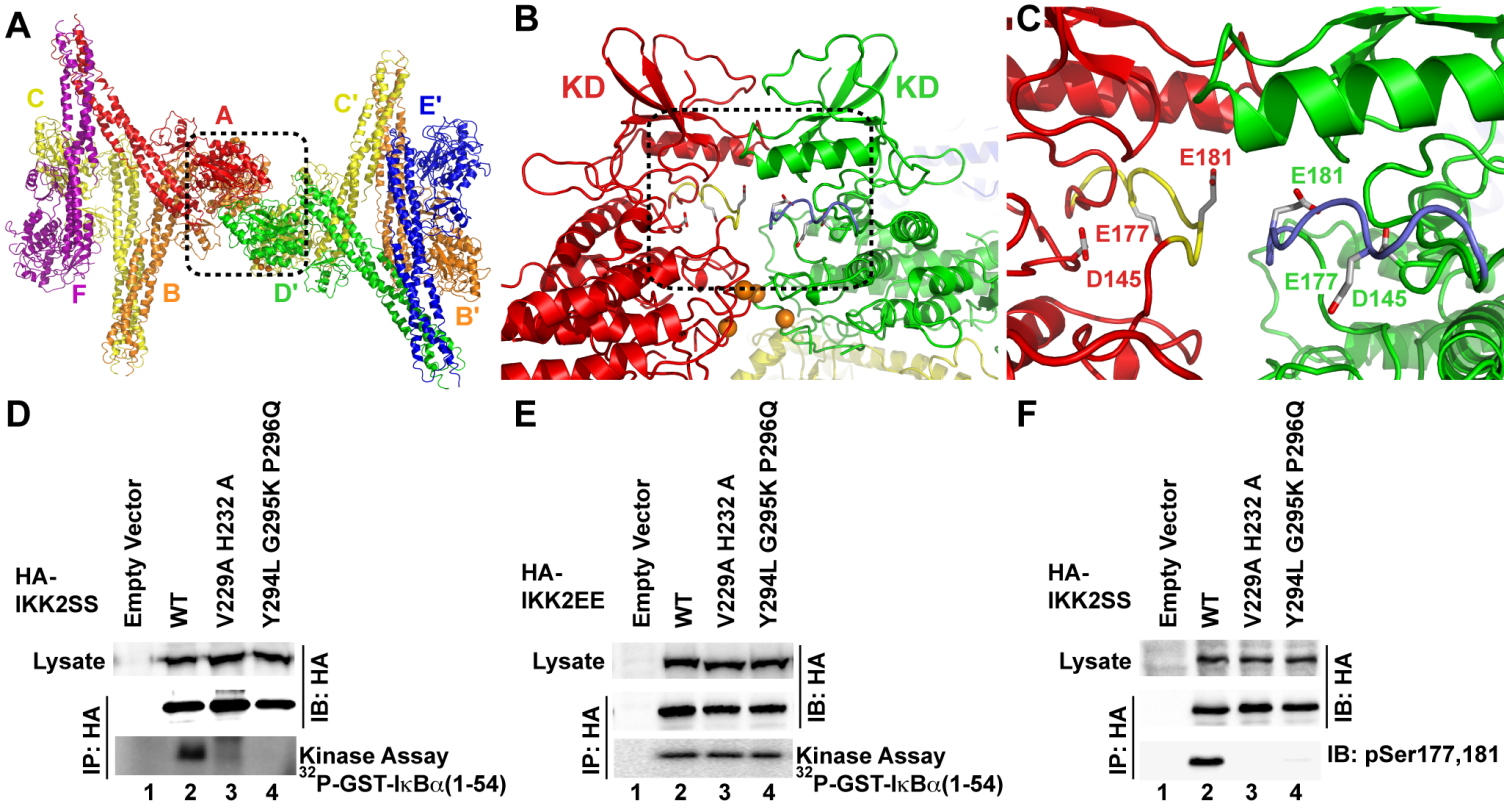
Interaction between KDs of oligomeric hIKK2. (A) Within the crystal, neighboring tetrameric assemblies interact symmetrically such that they contact one another through their V-shaped interfaces and two KDs are positioned within close proximity to one another (dashed box). (B) The close packed KDs are positioned so that their activation loops (dashed box) rest directly over the active site of a neighbor. Orange spheres mark the Cα positions on V229 and H232. (C) Close-up view of the kinase activation loops (yellow and blue) with glutamic acid residues 177 and 181 mimicking activation loop serines and the catalytic base D145 labeled. (D) In vitro kinase assay on immunoprecipitated hIKK2 with mutations at key residues that mediate KD–KD interactions in the crystal (lanes 3,4) reveals their involvement in catalytic activity. (E) Mutation of activation loop serines 177 and 181 to glutamates restores activity of immunoprecipitated IKK2 in vitro. (F) Immunoblotting with anti-phospho-Ser177,181 antibody reveals that the decrease in catalytic activity observed in the KD–KD interface mutants correlates with decreased activation loop phosphorylation.

We next wished to investigate the mechanism through which mutation of residues at the V-shaped interface blocks the transition of inactive hIKK2 to its active state. One possibility is suggested by the fact that oligomerization of hIKK2 dimers projects the KDs of the two subunits interacting through the V-shaped interface away from its dimer partner in a manner that could support KD–KD interactions. Indeed, further inspection of the arrangement of hIKK2 protomers in the crystal reveals that one of the largest crystal contact surfaces between symmetry-related units within the crystal occurs between four KDs at two V-shaped interfaces (Figure 7A). The orientation of the KDs at the V-shaped interface permits them to approach one another in a manner that brings the activation loop of one KD within close proximity to the active site of another, and vice versa. For example, E181 (the mutated phosphomimetic version of S181) from protomer A is positioned within only 5 Å of E181 from protomer D′ from a neighboring asymmetric unit in the crystal (Figure 7B,C). Only slight structural rearrangement would be required for the activation loop serines to insert into the active site of the opposing subunit for *trans* auto-phosphorylation.

In order to test whether these symmetrical KD–KD interaction surfaces are involved in the process of hIKK2 activation in cells, we produced mutant enzymes and tested their ability to become activated through in vitro kinase assays and immunoblot. Two mutants, V229A/H232A and Y294L/G295K/P296Q, displayed severe defects in their ability to phosphorylate an IκBα substrate after transfection and immunoprecipitation from transfected HEK293T cells (Figure 7D). In the triple mutant, three residues in hIKK2 at the supposed KD–KD interface were replaced with corresponding residues from hIKK1, suggesting that the activation mechanisms of IKK1 and IKK2 may differ. When the same mutations are introduced against the constitutively active S177E/S181E background and the resulting proteins are immunoprecipitated and assayed, we observe that they function with activity similar to the constitutively active native enzyme (Figure 7E). In order to determine whether the observed decrease in kinase activity of the KD–KD mutant enzymes results from an inability to adopt their active state, the phosphorylation status of transfected hIKK2 enzymes was probed by immunoblot with antiphosphoserine 177/181 antibody. Both KD–KD mutants displayed extremely low levels of activation loop phosphorylation relative to the native enzyme (Figure 7F). These experiments suggest that the surfaces observed to mediate KD–KD interactions in the hIKK2 crystal structure are necessary for *trans* auto-phosphorylation within the activation loop in cells. Interestingly, such a mechanism is supported by the recently published high-resolution X-ray crystal structure of the KD–ULD portion of the IKK-related TANK-binding Kinase 1 (TBK1). In that crystal structure, activation loop amino acids 164–199 of one TBK1 KD were repositioned such that the critical Ser172 phosphorylation site had entered the active site of the neighboring kinase [27].

**Figure 8:**
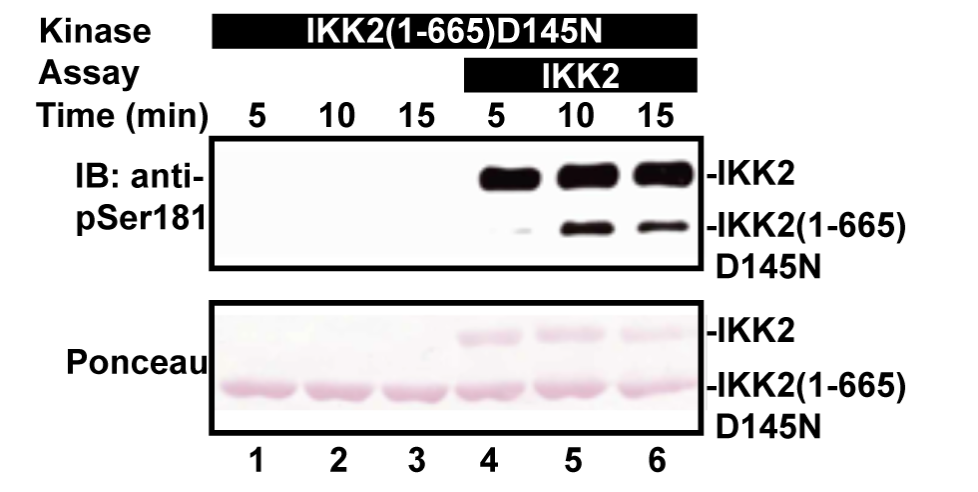
In vitro reconstitution of hIKK2 *trans* auto-phosphorylation. A catalytically inactive (D145N) and C-terminally truncated IKK2 (lanes 1–6) and mixtures of that enzyme with a catalytically active full-length version (lanes 4–6) were incubated with Mg-ATP for the time periods indicated and then probed via Western blot with anti-phosphoSer181 antibody (above) or by SDS PAGE (below).

In support of a mechanistic model whereby IKK2 becomes activated through oligomerization-dependent *trans* auto-phosphorylation, we next investigated whether IKK2 *trans* auto-phosphorylation could be reconstituted in vitro. To this end, we expressed and purified a C-terminally truncated version of hIKK2 in which the catalytic D145 residue was mutated to asparagine. The resulting protein is incapable of supporting phosphoryl group transfer, but can be phosphorylated on its activation loop serine residues. Immunodetection with an antibody specific for phosphorylated S181 of IKK2 confirmed that this mutant enzyme could be phosphorylated in *trans* on its activation loop upon incubation with native full-length hIKK2 enzyme and Mg-ATP (Figure 8).

### The Influence of NEMO on IKK2 Oligomerization

Our structure-based biochemical analyses suggest oligomerization-dependent *trans* auto-phosphorylation as a mechanism by which IKK2 can switch from its inactive form to a state of catalytic activity. Such a mechanism demands that there exist some manner of regulation to keep auto-phosphorylation from occurring constantly. We propose that fine regulation of IKK2 homo-oligomerization is part of the function of the obligatory IKK subunit NEMO. The IKK2–NEMO interaction has been well studied in vitro and in transfection systems [16],[20],[28]. Binding occurs at cellular steady state and requires small segments from the N-terminal portion of NEMO and the C-terminal NEMO-binding region of IKK2. Moreover, it has been widely believed that IKK2 switches from a low to a high molecular weight species upon cell stimulation.

**Figure 9:**
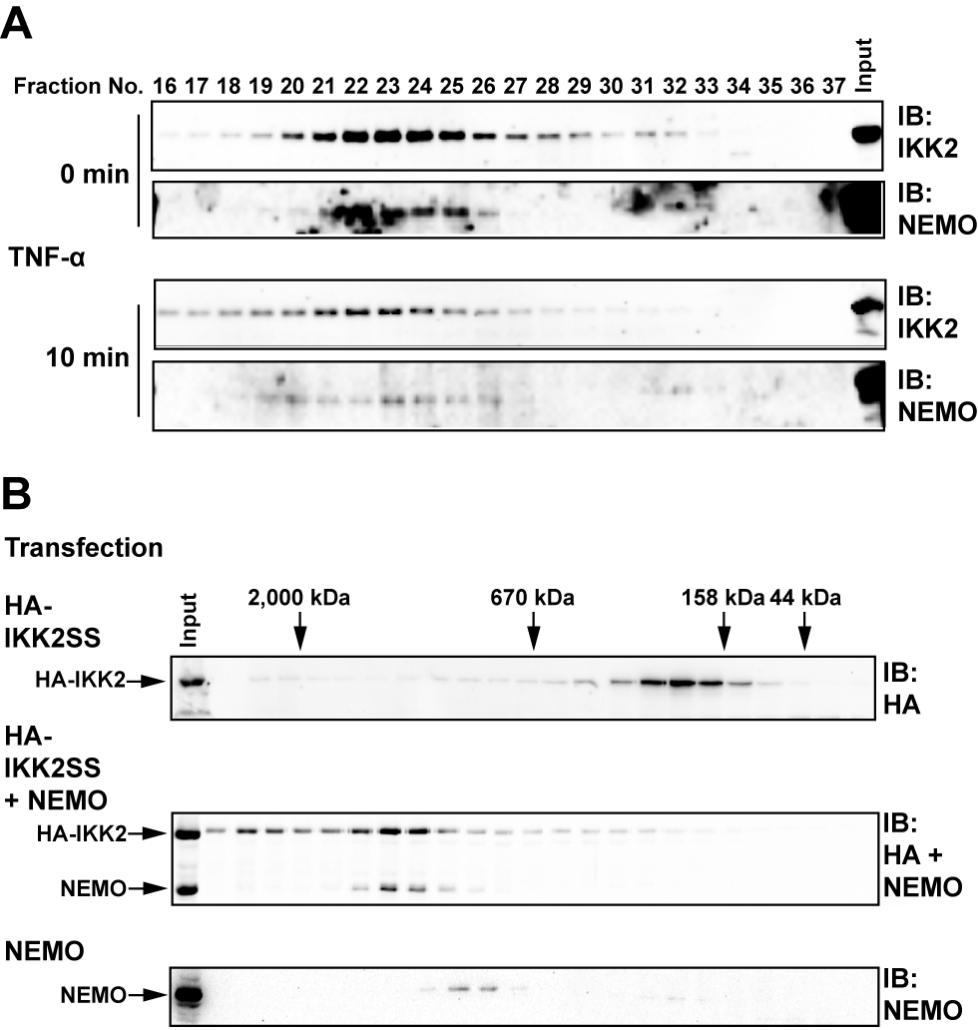
Size exclusion chromatography of endogenous and transfected IKK complexes. (A) Cytosolic extracts of untreated (above) and treated (below; 10 ng/mL of TNF for 10 min) MEF-3T3 cells were loaded onto a Superose 6 HR size exclusion column and fractionated at a flow rate of 0.5 mL/min. Fraction size was 0.5 mL. Fractions, as shown in the figure, were resolved on 10% SDS-PAGE and probed with anti-IKK2 and anti-NEMO antibodies. The experiment was performed at least thrice and representative blots are shown here. (B) HEK293T cells were transfected with HA-IKK2 (above), HA-IKK2 and NEMO (middle), or NEMO alone (below) and lysates were separated by size exclusion chromatography and probed by anti-HA or anti-NEMO antibodies.

In an effort to observe whether NEMO controls higher order oligomerization of IKK2, we carried out size exclusion fractionation of endogenous and transfected IKK2–NEMO complexes. Surprisingly, our studies of IKK2 oligomerization in cells disagree with the popularly held view. We observe that endogenous cellular IKK2–NEMO complexes elute at a high molecular weight both before and after TNF-α stimulation (Figure 9A). Moreover, transfected IKK2–NEMO complexes also exhibit comparably high molecular weight upon fractionation by size exclusion chromatography (Figure 9B).

## Discussion

### Oligomerization in the Process of IKK2 Activation

**Figure 10:**
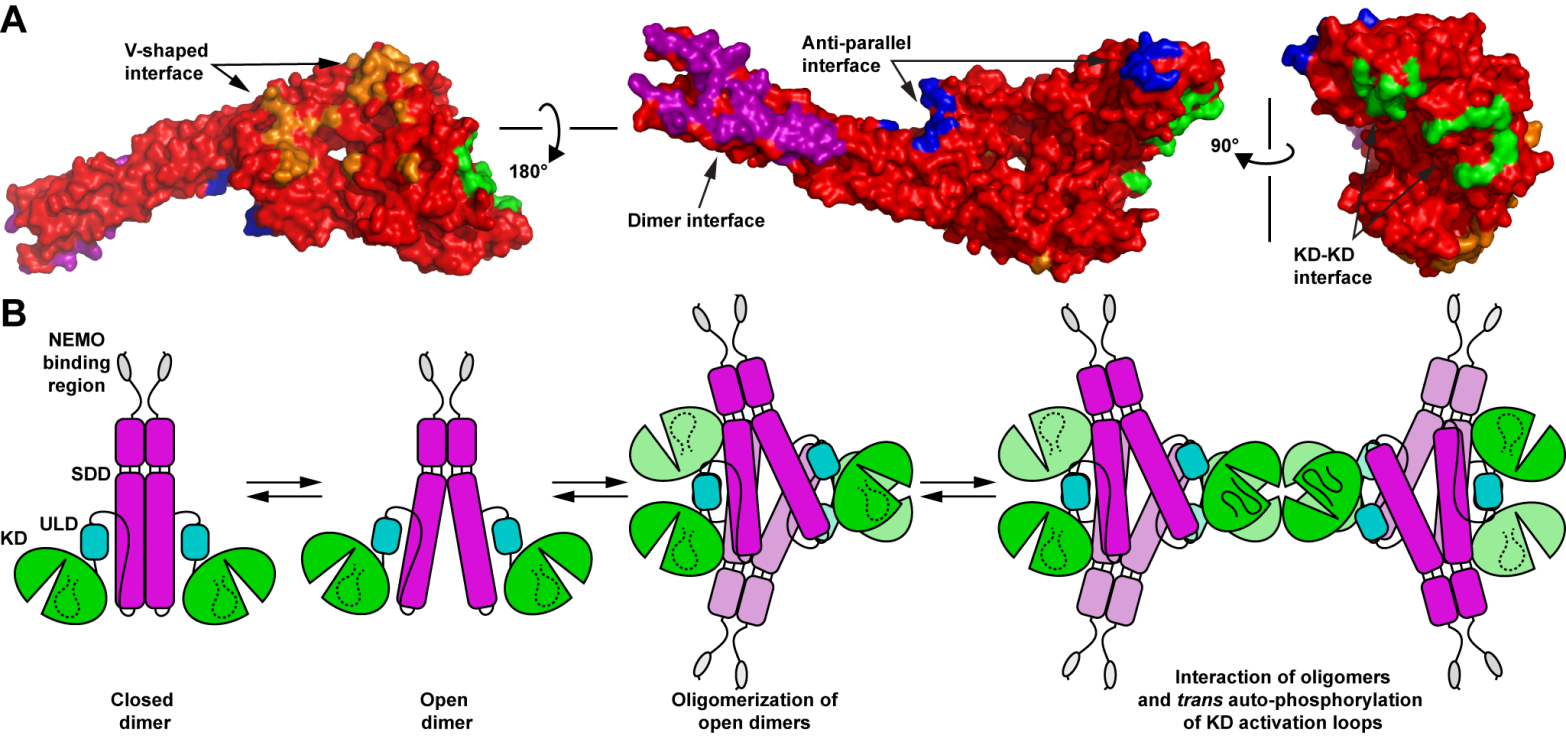
IKK2 oligomerization activation model. (A) The hIKK2 X-ray crystal structure in space filling representation viewed from three different angles. The four surfaces that mediate oligomerization in the X-ray crystal structure are colored purple (dimer interface), blue (antiparallel interface), orange (V-shaped interface), and green (KD–KD interface). (B) A structure-based model for IKK2 activation via *trans* auto-phosphorylation. IKK2 interconverts between its open and closed dimeric forms. The open dimer can further associate to form transient homooligomers, such as observed in the hIKK2 X-ray crystal structure. Phosphorylation of one IKK2 subunit by an upstream kinase activates the kinase activity of that subunit and, as a consequence of its propensity to assemble into higher order oligomers through it V-shaped and KD-KD interfaces, is rapidly amplified via *trans* auto-phosphorylation.

Our crystallographic and structure-based biochemical analyses of hIKK2 in its catalytically active conformation points to a model for kinase activation wherein dynamic association and dissociation of hIKK2 subunits occurs through multiple interfaces (Figure 10A). The predominant surface involved in association of individual IKK2 subunits is the distal SDD that mediates dimerization. Flexibility within the SDD permits a transition from the closed dimeric conformation observed in the xIKK2 crystal structure to the open conformation observed in hIKK2. This change in conformation is important as the active sites of two neighboring KDs cannot approach one another in the closed conformation without the two molecules clashing. In light of the fact that the hIKK2 KD exhibits an active conformation while the otherwise constitutively active xIKK2 KD was crystallized bound to a small molecule inhibitor compound, it is tempting to speculate that the transition between these two extreme dimeric conformations is linked to the activation state of the kinase. However, two recently published reports on X-ray crystal structures of the IKK-related protein TBK1 show that both active and inactive forms crystallize in varying degrees of the closed conformation [29],[30]. This suggests that the switch between open and closed conformation is transient even at concentrations of protein high enough to support crystal formation.

Additional portions of the IKK2 molecular surface are necessary for its activation as indicated by our structure-based cellular assays. One involves a face of the KD–ULD together with a central portion of the SDD to mediate the V-shaped interaction. Another is a broad region on the KD proximal to the activation loop that supports KD–KD interactions within the crystal. Combining these observations with those obtained previously from structural and biochemical investigation into xIKK2 leads to the emergence of a new structure-based mechanism for the conversion of IKK2 from inactive dimer to functioning kinase (Figure 10B). In this model, dimeric IKK2 alternates between closed and open conformations in the cell. Oligomerization of open IKK2 dimers through the V-shaped interaction observed in the X-ray crystal structures serves to stabilize the enzyme in a conformation that permits transient homotypic interactions through the IKK2 KD to support *trans* auto-phosphorylation (Figure S4).

This IKK2 oligomerization hypothesis helps explain the difficulty researchers have experienced in identifying a single upstream IKK2 kinase responsible for activating IKK2 by phosphorylating its activation loop S177 and S181 residues. Moreover, transient oligomerization of IKK2 in a conformation that promotes *trans* auto-phosphorylation would naturally direct and amplify the activity of any signaling kinase functioning directly upstream of IKK2 to quickly generate a pool of catalytically active IKK2, consistent with the rapid amplification of IKK2 phosphorylation that is observed in vivo. Finally, our discovery that the introduction of mutations within the oligomerization interfaces observed in the hIKK2 X-ray crystal structure is sufficient to disrupt activation suggests that small molecules designed to interfere with oligomerization through these interfaces should function as specific inhibitors of IKK2.

### The Integral Role of NEMO in IKK Activation

The biggest questions that remain regarding IKK2 activation involve the fundamental role played by NEMO. It has been clearly demonstrated that NEMO is central to NF-κB activation in response to classical pro-inflammatory stimuli [31]. Contrary to published reports, our data suggest that endogenous IKK2–NEMO complexes do not undergo a significant change in size upon TNF-α stimulation of cells. It is possible that difficulties in accurate measurement of IKK complex size are simply due to the elongated coiled-coil nature of NEMO, which consequently runs as a high molecular weight independent of IKK activation state and even when transfected and overexpressed relative to IKK2. However, an even greater problem appears to be the misinterpretation of published experimental data that clearly show a sharp increase in kinase activity, *though not necessarily the molecular mass*, of the high molecular weight IKK complex fraction upon stimulation of cells with pro-inflammatory stimuli.

The absence of a significant shift in molecular weight of the IKK2–NEMO complex upon cell stimulation and kinase activation suggests that IKK2 interaction through the oligomerization interfaces identified in our X-ray crystal structures is transient in nature. We propose that association with NEMO primes IKK2 so that it can become readily activated in response to early signaling events including receptor-mediated signalsome assembly and poly-ubiquitin chain formation. The precise mechanism by which NEMO prepares IKK2 for activation remains unclear, though it is apparently passive as the ability of NEMO to interact with IKK2 remains unchanged throughout the process of NF-κB induction. It is apparent, however, that the dependence upon NEMO for IKK2 activation can be circumvented by increasing the IKK2 concentration either in vitro or by its overexpression in transfected cells. This is why transfected IKK2–NEMO complexes are active while endogenous IKK2–NEMO remain inactive unless first induced by a pro-inflammatory stimulus [32]. We suggest that early signaling events involving poly-ubiquitin chains allow NEMO to permit rapid, transient oligomerization of IKK2, which leads directly to activation of the kinase catalytic activation via *trans* auto-phosphorylation. Such rapid binding and dissociation could then allow IKK2 activity to become amplified efficiently through activation loop *trans* auto-phosphorylation of neighboring transient assemblages. This model agrees well with a recently published report that free poly-ubiquitin chains can induce IKK activation via *trans* auto-phosphorylation [21]. NEMO also plays additional roles in facilitating NF-κB activation, such as by directing IKK2 catalytic activity toward IκBα via interaction with the NF-κB inhibitor through its C-terminal zinc finger domain [33].

## Materials and Methods

### Plasmids, Oligonucleotides, and Cell Lines

The full-length hIKK2 cDNA clone was graciously provided by Y. Chen and the laboratory of M. Karin. For large-scale purification and in vitro biochemical assays, full-length human IKK2 was cloned in pFastBacHTb (Invitrogen) vector within BamHI and NotI sites giving rise to an N-terminal hexa-Histidine tag followed by TEV protease recognition sequence. Codons corresponding to S177 and S181 were mutated to E by the Quickchange mutagenesis protocol (Agilent) to generate the hIKK2EE clone. The hIKK2EE 1–700 construct was created by mutating hIKK2 amino acid 701 to a stop codon. Gene fragments corresponding to hIKK2EE 11–669 and 11–700 were amplified by PCR and subcloned into pFastBacHTb. For cellular assays, hIKK2 was cloned as a HA-tagged version into pRC or pCMV-HA (Clontech) vector. For ex vivo cellular biochemistry assays, HA-IKK2 constructs were transfected into HEK293T cells using Lipofectamine 2000 (Invitrogen) reagent following the manufacturer's protocol. All oligonucleotides were purchased from IDT. Restriction enzymes and T4 DNA ligase were procured from New England Biolabs.

### Protein Expression and Purification

Recombinant virus production and protein expression were performed with modifications of a previously published protocol [26]. Sf9 suspension cultures were infected with baculoviruses at a cell density of 2×10^6^/mL and allowed to grow for 48–72 h postinfection. Cells were harvested and lysed in Lysis buffer (25 mM Tris pH 8.0, 200 mM NaCl, 10 mM imidazole, 10% Glycerol, 5 mM β-Mercaptoethanol, and protease inhibitor cocktail) by sonication. The lysate was clarified by centrifugation at 14,000 rpm for 45 min at 4°C and loaded onto Ni-NTA resin (Qiagen). The resin was thoroughly washed with Lysis buffer containing 30 mM Imidazole prior to elution using the same buffer with 250 mM imidazole. Peak fractions were pooled and treated with TEV protease to remove the His-tag. The protein was then incubated with 1 mM ATP in presence of 20 mM MgCl_2_, 20 mM β-glycerophosphate, 10 mM NaF, and 1 mM sodium orthovanadate for 1 h and loaded onto preparative Superdex200 size exclusion column connected to an ÄKTA purifier (GE Healthcare) equilibrated with 20 mM Tris-HCl (pH 8.0), 150 mM NaCl, 5 mM DTT, 5% glycerol. Peak fractions were concentrated by centrifugation using 30 kDa cutoff membrane concentrator unit (Millipore). Protein concentration was determined using Bradford reagent (BioRad) and was snap frozen in liquid N_2_ for long-term storage at −80°C. GST-IκBα1-54 was purified from *E. coli* BL21(DE3) cells using Glutathione-Sepharose (GE Healthcare) resin.

### Crystallization

Both crystal forms were obtained by hanging drop vapor diffusion method. Protein sample was mixed with Inhibitor MLN120B (Millennium Pharmaceuticals) at a molar ratio of 1∶2 and incubated for 30 min. We combined 1 µL of protein–inhibitor mixture with an equal amount of reservoir solution containing 1.5 to 2 M sodium malonate pH 6.0, 0.3–0.5 M ammonium acetate, and 10 mM DTT and equilibrated over the same solution at 18 or 20°C. Hexagonal crystal appeared within 2–3 d and grew to maximum dimension of 50×50×400 µm within 10–14 d. The crystals were frozen in liquid N_2_ using well solution containing 25%–30% Glycerol or 2.5 M sodium malonate as cryoprotectant. Tetragonal crystals were obtained by mixing equal volumes of protein–inhibitor complex with 1.0–1.3 M sodium malonate pH 6.5, 0.2–0.4 M Ammonium acetate, and 10 mM DTT and equilibrating the drop over the same solution at 18°C. Crystals appeared in 15 d and took nearly a month to grow to a final size of up to 70×70×250 µm. Crystals were cryo-preserved in a similar fashion mentioned above and stored in liquid N_2_ prior to data collection.

### X-Ray Diffraction Data Collection

X-ray diffraction data were collected on a Quantum 315 CCD detector (ADSC) at the Advanced Photon Source synchrotron beamline 19ID at Argonne National Laboratory. The hexagonal crystal form gave strong diffraction patterns that failed to extend beyond 6.95 Å resolution. The diffraction pattern of the tetragonal crystal extended to 4 Å and revealed that the crystal belongs to space group P4_1_22 or P4_3_22 with unit cell: a = b = 170.81, c = 509.56 Å. Diffraction intensities were indexed, integrated, and scaled using HKL2000 [34]. Data collection statistics are summarized in Table 1 and Table S1.

### Structure Solution

Due to data quality and resolution and the presence of multiple molecules in the asymmetric unit, molecular replacement proved challenging. Initially, a brute force molecular replacement strategy was employed in which more than one hundred different IKK2 tetramer, dimer, or monomer models were generated manually based on the xIKK2 structure and used as search probes in various software packages. One of these test models identified two pairs of closely packed protomers (chains A:B and C:D) in space group P4_1_22 using the CCP4 program MOLREP [35]. With the A:B and C:D dimers fixed, one rigid-body refined monomer was then used as search model in MOLREP to identify the position of chain E, which participates in a similar interaction with its own symmetry related partner along a 2-fold axis. Crystal packing and electron density map calculation from this initial model indicated that there were likely additional molecules in the asymmetric unit but molecular replacement failed to locate them. The initial position of the sixth molecule, monomer F, was obtained by rotating monomer E about the 2-fold pseudo-symmetry axis relating the A:B and C:D V-shaped dimers. Refinement and crystal packing confirmed this to be the correct position for the sixth IKK2 subunit in the asymmetric unit. With six molecules in the asymmetric unit, the calculated solvent volume in the crystal is 64%.

### Model Building and Refinement

The orientation and position of the initial model containing six IKK2 chains were refined by rigid-body refinement followed by minimization and simulated annealing refinement with a maximum likelihood target function and a flat bulk-solvent correction using the CNS system version 1.1 [36]. Model rebuilding was carried out using 2*F*_O_-*F*_C_ electron density maps in XTALVIEW [37]. Due to the relatively low resolution data used throughout refinement, upon refinement of the model to *R*-factors of 27.5% (*R*-cryst) and 36.6% (*R*-free), CNS version 1.3 was employed in order to carry out Deformable Elastic Network (DEN)–assisted refinement [23]. Initially, coordinates from a rigid-body refined xIKK2 model were employed as a reference. After a few refinement cycles with application of strict NCS restraints, the hIKK2 exhibited better model geometry than the reference structures and the chain with the best geometry among the six molecules was used to direct building and refinement of the final crystallographic model. DEN refinement dramatically improved the geometry and *R*-factors of the hIKK2 structure to a final *R*-cryst of 26.7% and *R*-free of 29.9%. Detailed refinement statistics are included in Table 1. Coordinates and structure factors for the hIKK2(11-669)EE crystal structure have been deposited into the Protein Data Bank as entry code 4E3C [38]. Model figures were made in PyMOL [39].

### Immunoprecipitation and Kinase Assay

Following transfection, HEK293 cells were grown for 24 h before harvesting and lysing in RIPA buffer supplemented with 1× phosphatase inhibitor cocktail (Sigma). HA-IKK2 was immunoprecipitated by adding 1 µg of anti-HA antibody (Covance, 16B12) to each pre-cleared cell lysate and incubated for 2 h at 4°C, and protein G sepharose (GE Healthcare) was subsequently added to each sample and allowed to bind for another 1 h at 4°C with mixing. Beads were then precipitated and washed rigorously using the cytoplasmic extraction buffer (10 mM HEPES–KOH pH 7.9, 250 mM NaCl, 1 mM EDTA, 0.5% NP-40, 0.2% Tween 20, 2 mM DTT, 1 mM PMSF, 20 mM β-glycerophosphate, 10 mM NaF, 0.1 mM Sodium orthovanadate). Beads were washed with kinase assay buffer (20 mM HEPES–KOH pH 7.7, 100 mM NaCl, 10 mM MgCl_2_, 2 mM DTT, 20 mM β-glycerophosphate, 10 mM NaF, 0.1 mM Sodium orthovanadate, 20 µM ATP) without ATP prior to kinase assay. Kinase assay was performed with immunoprecipitated IKK2 using GST-IκBα (1–54) as the substrate in presence of 10 µCi γ-^32^P-ATP at 30°C for 30 min. Kinase reactions were stopped by boiling with 1× Laemmli buffer and resolved on 10% SDS-PAGE. The gel portion containing radioactive substrate was excised and detected by autoradiography. The portion containing IKK2 was transferred to nitrocellulose membrane and probed with anti-HA antibody.

### Analytical Ultracentrifugation

Full-length His-tagged IKK2 and IKK2EE(11–669) were purified and prepared as previously described. Sedimentation velocity (SV) experiments were performed in a ProteomeLab XL-I (BeckmanCoulter) analytical ultracentrifuge. Protein samples were loaded at a concentration of ∼0.5 mg/ml in two-channel cells and spun in an An-50 Ti 8-place rotor at 30,000 rpm, 20°C for 15 h. Data were analyzed using Sedfit software (P. Schuck, NIH/NIBIB).

### SEC-MALLS

Full length His-tagged IKK2 was purified as previously described and treated with 1 mM ATP in the presence of 20 mM MgCl_2_ and 1 mM Na_3_VO_4_ prior to purification through a Superdex 200 16/60 column (GE Healthcare). Protein fractions were then concentrated in 30 kDa cutoff Amicon Ultra centrifugal concentrators (Millipore) to the indicated concentrations. We injected 20 µL of sample into a pre-equilibrated (20 mM Tris-Cl pH 8, 150 mM NaCl, 5% w/v glycerol) Zenix SEC-300, 7.8×300 mm column (Sepax Technologies, Inc.) at 1 mL/min. Elution of the protein was monitored by UV-visible (Shimadzu SPD-10A VP) and refractive index (Hitachi L-2490) detectors. Light scattering was monitored with a Dawn–Helios multi-angle detector (Wyatt technology). Astra VI software (Wyatt Technology) was used to analyze light scattering data.

### In Vitro *Trans* Auto-Phosphorylation Kinase Assay

Full-length His-tagged IKK2 and the C-terminal truncated catalytically inactive mutant IKK2(1–665)D145N were expressed in insect cells and purified as previously described. The two proteins were incubated together at 30°C in the presence of 20 µM ATP in a reaction buffer containing 20 mM Tris-HCl pH 7.5, 100 mM NaCl, 2 mM DTT, 1 mM Na_3_VO_4_, 10 mM NaF, 20 mM β-glycerophosphate, and 10 mM PNPP. Reactions were halted at the indicated times by adding 1× Laemmli buffer and heating the sample at 95°C for 5 min. Samples were resolved on 8% SDS-PAGE and analyzed by immunoblot using antibody against phospho-Ser181 of IKK2 (Cell Signaling Technology).

### Fractionation by Size Exclusion Chromatography

Cytoplasmic extracts of HEK293T cells transfected with HA-IKK2 with or without NEMO were prepared. We loaded 200 µl of these extracts onto Superose 6 GL10/300 (GE Healthcare) column equilibrated with 25 mM Tris pH 7.5, 150 mM NaCl, 5% glycerol, 2 mM DTT connected to an ÄKTA system. We collected 0.5 mL fractions at a flow rate of 0.5 mL/min, and 15 µL of samples were loaded onto SDS-PAGE and analyzed by WB using anti-HA and anti-NEMO antibodies. 3T3 cells were stimulated with 10 ng/mL rhTNF (Roche) for 10 min. Cells were lysed in buffer containing 10 mM HEPES, 1.5 mM MgCl_2_, 10 mM KCl, 0.5% NP-40, 0.5 mM DTT. We fractionated 0.5 mg of lysate with a Superose 6 GL10/300 (GE Healthcare) column using a buffer containing 25 mM Tris pH 7.5, 150 mM NaCl, 5% glycerol, 2 mM DTT in an ÄKTA system. 0.5 mL fractions were collected and analyzed by western blotting using anti-IKK2 (Millipore) or anti-NEMO (Santa Cruz Biotechnology) antibodies.

## Supporting Information

Figure S1 Primary sequence alignment of IKK subunit proteins. Human IKK2 (hIKK2) and IKK1 (hIKK1) proteins are aligned with murine IKK2 (mIKK2) and IKK1 (mIKK1) as well as the IKK2 protein from *Xenopus laevis* (xIKK2). The human cyclic-AMP-dependent protein kinase catalytic subunit KD (PKA) is also included in the alignment. Each sequence is numbered and tabs beneath stacked sequences mark every 10 amino acids in hIKK2 sequence. Identities are boxed in black, conserved positions are dark grey, and homologous substitutions are light grey. Secondary structure elements in the hIKK2 crystal structure are depicted as arrows (beta strands) and cylinders (alpha helices) above their corresponding residues in the primary sequence and are named. Portions of the structure for which there was no electron density are indicated as dashed lines. The colors of the secondary structure elements represent the three domains of hIKK2 as defined in Figure 1A,B (KD, green; ubiquitin-like domain, cyan; scaffold dimerization domain, magenta). Amino acid residues that participate in interdomain interactions with the KD are indicated by green circle, a cyan circle marks residues that contact the ubiquitin-like domain, and magenta circles designate residues that contact the scaffold dimerization domain (contact distance of <4.9 Å as calculated from chain B). The “•” symbol marks amino acid residues that are involved in dimerization (calculated from chains B and C); “v” marks residues that mediate the V-shaped interface (taken from chains A and B); “ = ” corresponds to residues at the antiparallel interface (calculated from chains C and F); and “x” identifies residues that contact one another at the interface of two KDs at the crystallographic axis (taken from chains A and D′).
(TIF)

Figure S2 (A) A portion of the hIKK2EE 2*F*_O_-*F*_C_ electron density map that covers the KD of subunit C. The model, oriented as in Figure 2C, is shown as yellow backbone sticks and the electron density contoured at 1.1 σ is in cyan. (B) Close-up view of the activation loop of hIKK2EE from the KD of subunit C. Electron density contoured at 1.1 σ is in cyan and the model is shown as sticks with several side chains, including the S177E mutation, labeled. This region is representative of the electron density throughout the unit cell. (C) Superposition of the KDs and key catalytic residues from hIKK2 and cAMP-dependent protein kinase (PKA) in their active conformations. Residues that form the “hydrophobic spine” of PKA are depicted as space-filling spheres (yellow for hIKK2; orange for PKA) and their numbering (bright pink) is for PKA. Additional side chains are depicted as sticks and labeled (purple) with hIKK2 numbering.
(TIF)

Figure S3 X-ray structure of hIKK2 from a hexagonal crystal form. (A) The domain organization of an hIKK2 construct containing amino acids 1–700 with serine residues 177 and 181 mutated to glutamic acid is shown schematically in comparison to the native human protein. (B) Rigid-body refinement of the molecular replacement solution yields an hIKK2 dimer with an open configuration, similar to that observed in the tetragonal crystal form. The two subunits are depicted in different shades of green in ribbon diagram (above) and surface (below) representations. (C) Crystal packing of the hIKK2EE(1–700) in the hexagonal crystal reveals that the dimer asymmetric unit employs the same V-shaped and antiparallel interfaces in order to assemble. One half of one unit cell is depicted with each of six cyrstallographically equivalent dimers represented in different colors. (D) Close-up view of two interacting dimers in the hexagonal unit cell. Note the similarity in the interactions between the two crystallographically equivalent dimers in this structure and the four versions of a similar arrangement observed in the tetragonal crystal form (Figure 4B).
(TIF)

Figure S4 The IKK2 oligomerization model of kinase activation. Ribbon diagram structures taken from the inactivated Xenopus IKK2 X-ray structure (above left) and the active human IKK2 X-ray crystal structure (others) represent different conformational and oligomeric forms of IKK2 as it is converted to its active state via opening of the dimer interface, induced proximity with neighboring dimers, and transient oligomerization in a conformation that promotes association between KDs and *trans* auto-phosphorylation.
(TIF)

Table S1 Data collection statistics for hexagonal IKK2EE(1-700) crystal.
(DOC)

## Abbreviations

ATP: adenosine-5′-triphosphate
AUC: analytical ultracentrifugation
DEN: deformable elastic network
IκB: inhibitor of NF-κB
hIKK2: human IKK2/IKKβ subunit
IKK: IκB kinase
KD: kinase domain
NEMO: NF-κB essential modulator
NF-κB: nuclear factor of κ light chain gene enhancer in B cells
SDD: scaffold dimerization domain
SEC-MALLS: size exclusion chromatography multi-angle laser light scattering
TAK1: transforming growth factor β activated kinase 1
TBK1: TANK-binding kinase 1
TEV: tobacco etch virus
ULD: ubiquitin-like domain
xIKK2: *Xenopus laevis* IKK2/IKKβ subunit
