# Supplementary figures and images for: A Structural Basis for IκB Kinase 2 Activation Via Oligomerization-Dependent Trans Auto-Phosphorylation
Source: PLoS Biol. 2013 Jun 11;11(6):e1001581. doi: 10.1371/journal.pbio.1001581 (PMC3678999; doi:10.1371/journal.pbio.1001581)

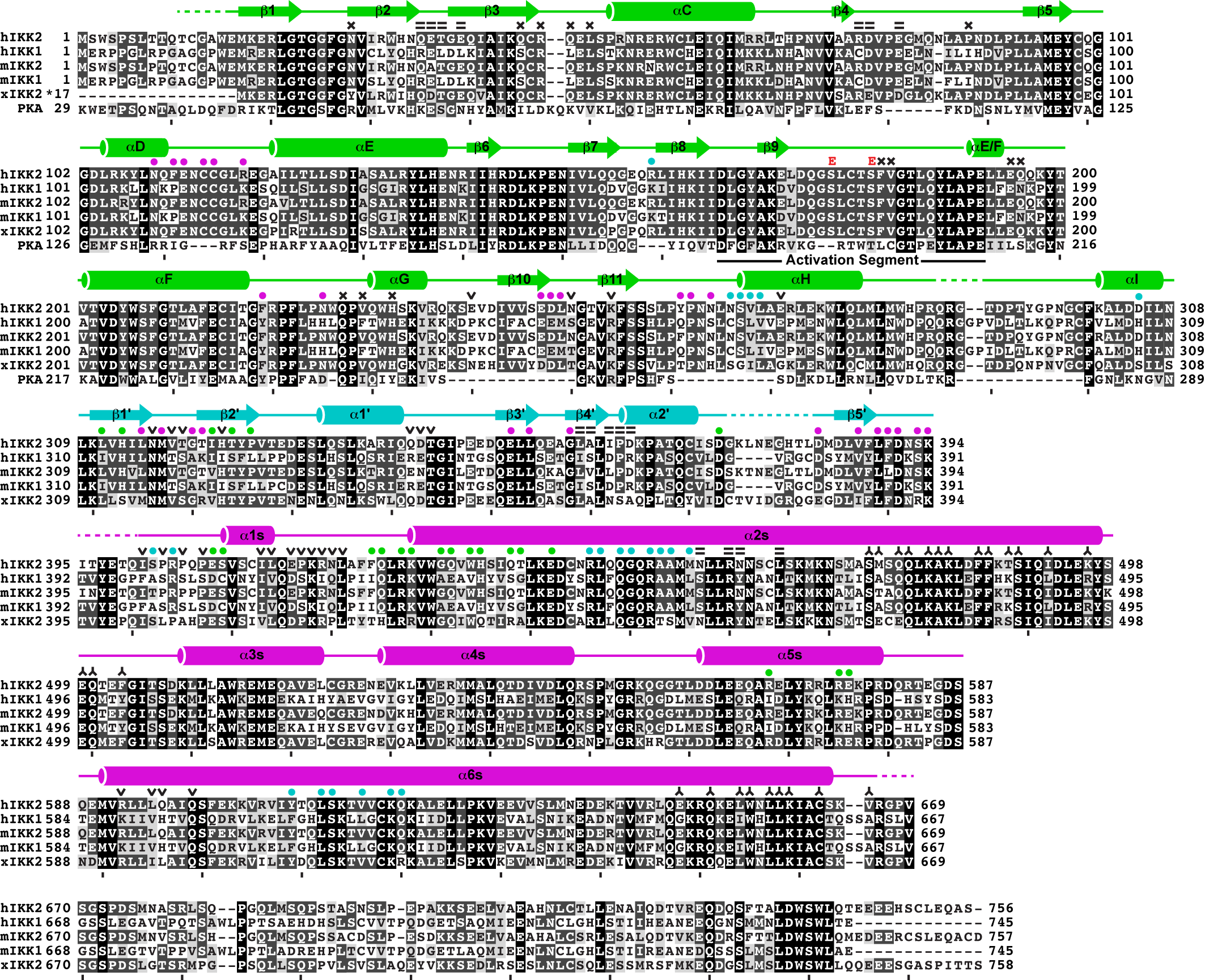

Supplement: Figure S1 — Primary sequence alignment of IKK subunit proteins. Human IKK2 (hIKK2) and IKK1 (hIKK1) proteins are aligned with murine IKK2 (mIKK2) and IKK1 (mIKK1) as well as the IKK2 protein from Xenopus laevis (xIKK2). The human cyclic-AMP-dependent protein kinase catalytic subunit KD (PKA) is also included in the alignment. Each sequence is numbered and tabs beneath stacked sequences mark every 10 amino acids in hIKK2 sequence. Identities are boxed in black, conserved positions are dark grey, and homologous substitutions are light grey. Secondary structure elements in the hIKK2 crystal structure are depicted as arrows (beta strands) and cylinders (alpha helices) above their corresponding residues in the primary sequence and are named. Portions of the structure for which there was no electron density are indicated as dashed lines. The colors of the secondary structure elements represent the three domains of hIKK2 as defined in Figure 1A,B (KD, green; ubiquitin-like domain, cyan; scaffold dimerization domain, magenta). Amino acid residues that participate in interdomain interactions with the KD are indicated by green circle, a cyan circle marks residues that contact the ubiquitin-like domain, and magenta circles designate residues that contact the scaffold dimerization domain (contact distance of <4.9 Å as calculated from chain B). The “•” symbol marks amino acid residues that are involved in dimerization (calculated from chains B and C); “v” marks residues that mediate the V-shaped interface (taken from chains A and B); “ = ” corresponds to residues at the antiparallel interface (calculated from chains C and F); and “x” identifies residues that contact one another at the interface of two KDs at the crystallographic axis (taken from chains A and D′). (TIF) [file pbio.1001581.s001.tif]

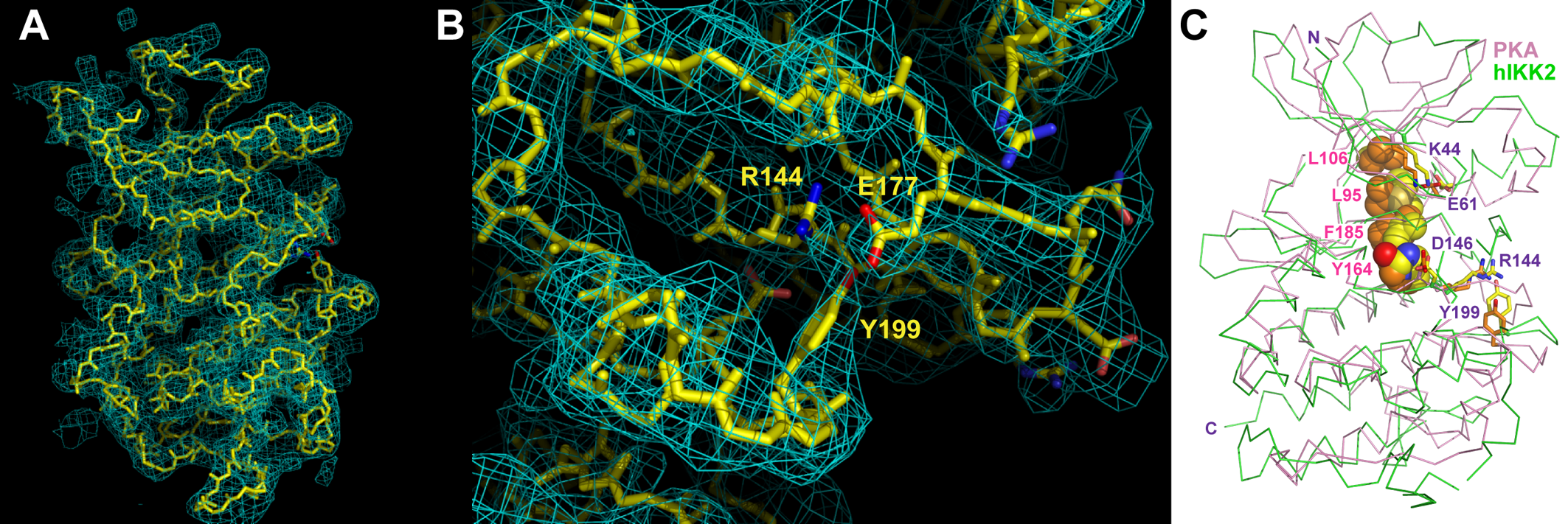

Supplement: Figure S2 — (A) A portion of the hIKK2EE 2F O-F C electron density map that covers the KD of subunit C. The model, oriented as in Figure 2C, is shown as yellow backbone sticks and the electron density contoured at 1.1 σ is in cyan. (B) Close-up view of the activation loop of hIKK2EE from the KD of subunit C. Electron density contoured at 1.1 σ is in cyan and the model is shown as sticks with several side chains, including the S177E mutation, labeled. This region is representative of the electron density throughout the unit cell. (C) Superposition of the KDs and key catalytic residues from hIKK2 and cAMP-dependent protein kinase (PKA) in their active conformations. Residues that form the “hydrophobic spine” of PKA are depicted as space-filling spheres (yellow for hIKK2; orange for PKA) and their numbering (bright pink) is for PKA. Additional side chains are depicted as sticks and labeled (purple) with hIKK2 numbering. (TIF) [file pbio.1001581.s002.tif]

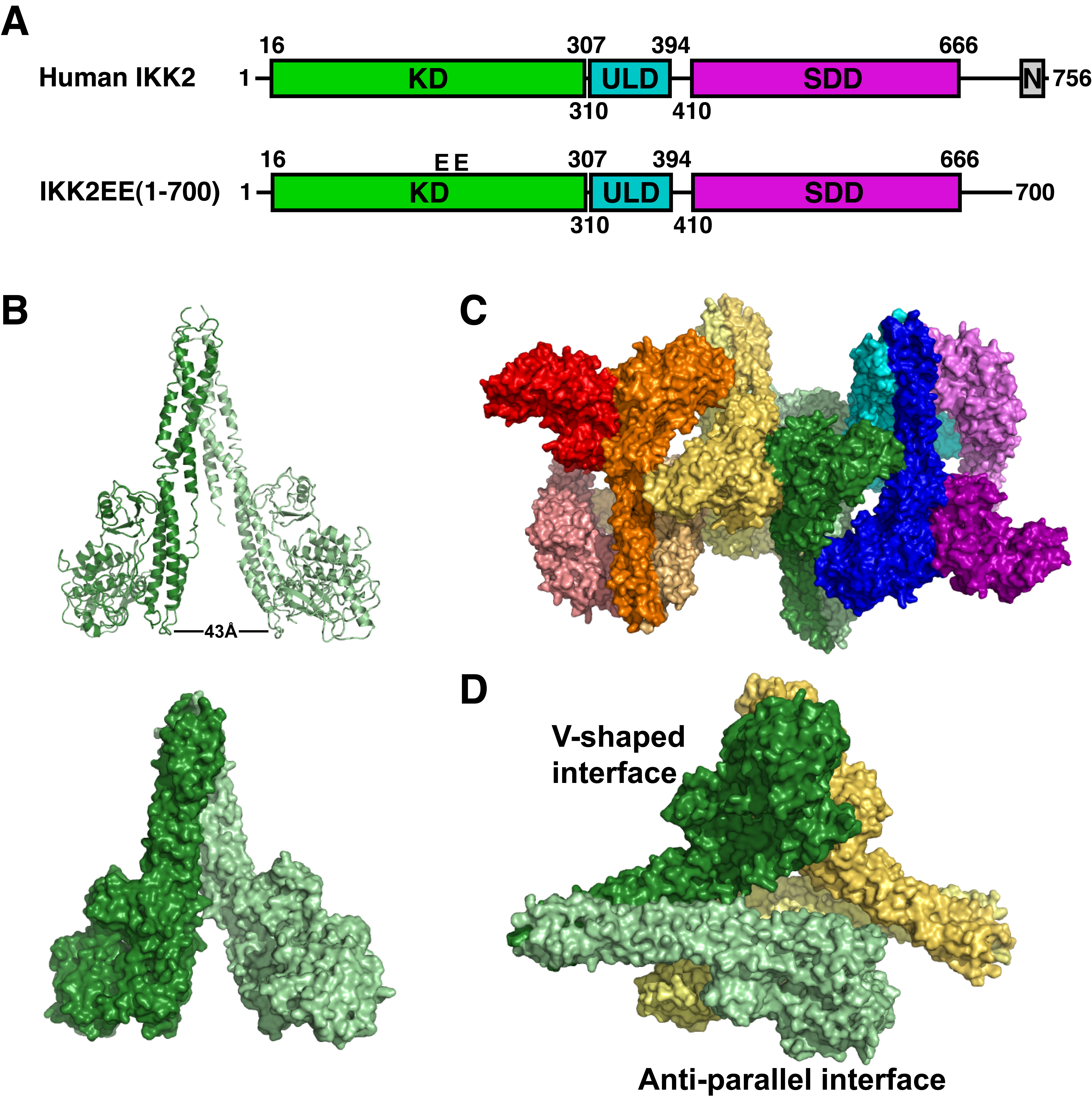

Supplement: Figure S3 — X-ray structure of hIKK2 from a hexagonal crystal form. (A) The domain organization of an hIKK2 construct containing amino acids 1–700 with serine residues 177 and 181 mutated to glutamic acid is shown schematically in comparison to the native human protein. (B) Rigid-body refinement of the molecular replacement solution yields an hIKK2 dimer with an open configuration, similar to that observed in the tetragonal crystal form. The two subunits are depicted in different shades of green in ribbon diagram (above) and surface (below) representations. (C) Crystal packing of the hIKK2EE(1–700) in the hexagonal crystal reveals that the dimer asymmetric unit employs the same V-shaped and antiparallel interfaces in order to assemble. One half of one unit cell is depicted with each of six cyrstallographically equivalent dimers represented in different colors. (D) Close-up view of two interacting dimers in the hexagonal unit cell. Note the similarity in the interactions between the two crystallographically equivalent dimers in this structure and the four versions of a similar arrangement observed in the tetragonal crystal form (Figure 4B). (TIF) [file pbio.1001581.s003.tif]

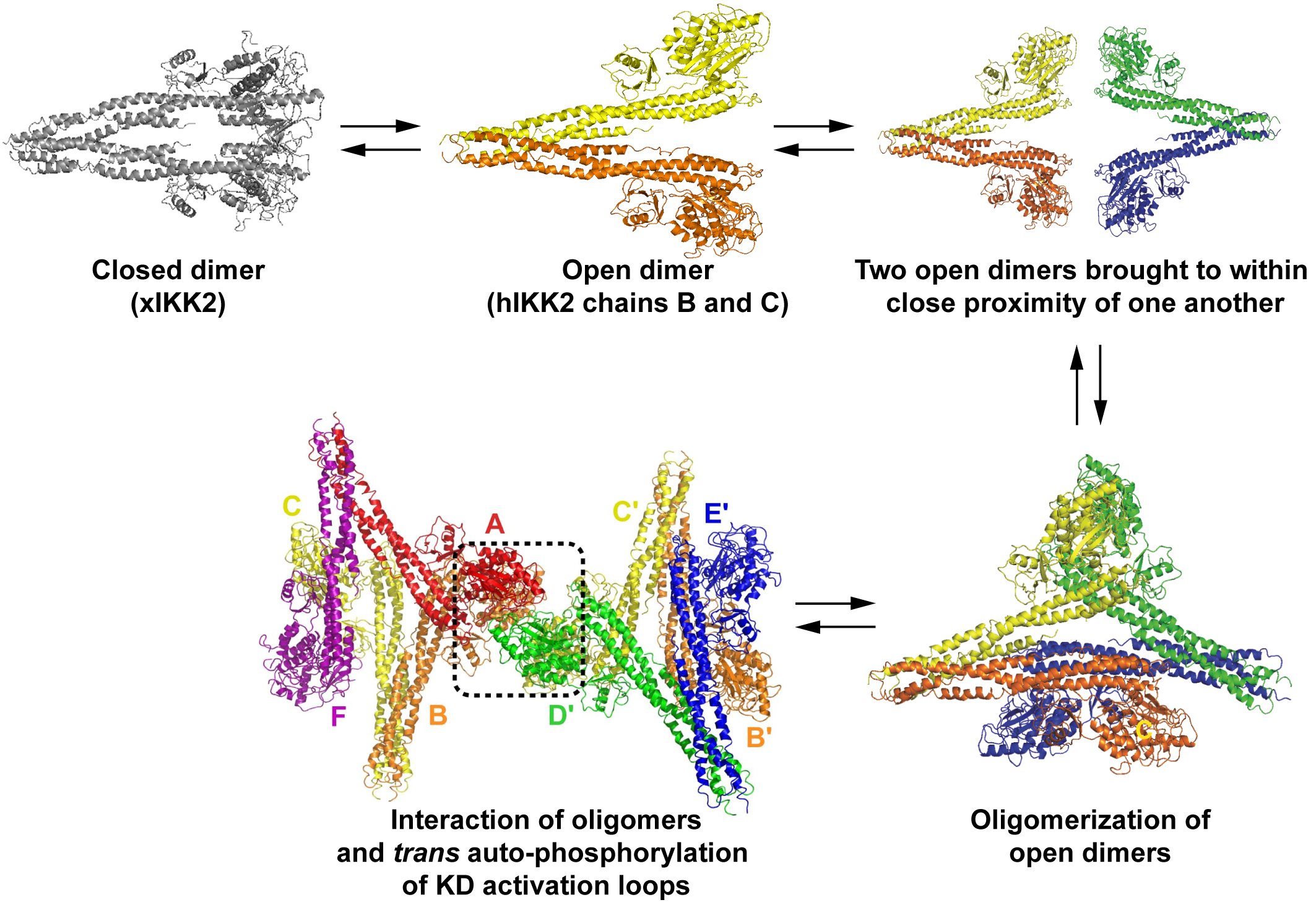

Supplement: Figure S4 — The IKK2 oligomerization model of kinase activation. Ribbon diagram structures taken from the inactivated Xenopus IKK2 X-ray structure (above left) and the active human IKK2 X-ray crystal structure (others) represent different conformational and oligomeric forms of IKK2 as it is converted to its active state via opening of the dimer interface, induced proximity with neighboring dimers, and transient oligomerization in a conformation that promotes association between KDs and trans auto-phosphorylation. (TIF) [file pbio.1001581.s004.tif]
