# Supplementary material for: A Structural Basis for IκB Kinase 2 Activation Via Oligomerization-Dependent Trans Auto-Phosphorylation
Source: PLoS Biol. 2013 Jun 11;11(6):e1001581. doi: 10.1371/journal.pbio.1001581 (PMC3678999; doi:10.1371/journal.pbio.1001581)
Supplement: Table S1 — Data collection statistics for hexagonal IKK2EE(1-700) crystal. (DOC) [file pbio.1001581.s005.doc]

| **Table S1.** Data collection statistics | |
| --- | --- |
|  | IKK2EE(1-700) |
| *Data collection* |  |
| X-ray source | APS 24ID |
| Wavelength (Å) | 0.97910 |
| Space group | P6_1_22 |
| Unit cell (Å) |  |
| a | 124.72 |
| b | 124.72 |
| c | 477.00 |
| Molecules/asymm. unit | 2 |
| Resolution range (Å)^1^ | 30.0-6.95(7.20-6.95) |
| *R*_sym_ (%) | 10.4(66.9) |
| Observations | 33592 |
| Unique reflections | 6704 |
| Completeness (%) | 99.9(100) |
| <*I*/σ> | 11.7(3.0) |
| ^1^Data in parentheses are for highest resolution shell | |
